# Bibliometric and visual analysis in the field of macrophages in Traditional Chinese Medicine from 2003 to 2023

**DOI:** 10.3389/fimmu.2025.1558926

**Published:** 2025-04-02

**Authors:** Wenxin Zhang, Kaidi Zhao, Ruimin Ma, Minghan Ma, Yuqiong Du, Peng Fang

**Affiliations:** ^1^ School of Traditional Chinese Medicine, Capital Medical University, Beijing, China; ^2^ College of Traditional Chinese Medicine, Shandong Second Medical University, Weifang, Shandong, China; ^3^ Department of Infectious Diseases, The First Affiliated Hospital of Zhejiang Chinese Medical University (Zhejiang Provincial Hospital of Chinese Medicine), Hangzhou, Zhejiang, China

**Keywords:** macrophages, bibliometrics, Traditional Chinese Medicine, VOSviewer, CiteSpace

## Abstract

**Objective:**

Macrophages are increasingly recognized as crucial therapeutic targets in the treatment of diverse pathological conditions. While considerable research has focused on macrophage-related mechanisms within Traditional Chinese Medicine (TCM), there remains a notable absence of comprehensive quantitative analyses in this field. This study aims to examine the evolutionary trajectory of macrophage-related research in TCM from 2003 to 2023, providing insights to guide future investigative directions.

**Methods:**

We searched for articles published between 2003 and 2023 from the Web of Science Core Collection (WoSCC) database and analyzed them using R software, VOSviewer and CiteSpace.

**Results:**

A total of 1,823 documents were obtained through the search. The results indicated that the number of publications between 2003 and 2023 exhibited an upward trend, with the majority of these documents originating from Chinese academic institutions and authored by Chinese scholars. This observation suggests a potential correlation with the growing prominence of Chinese medicine within China. Macrophage polarizations, a prominent focus in the study of macrophages, has also assumed an increasingly significant role in the domain of macrophages in TCM-related disciplines. The publication of these results also suggests that targeting macrophages in TCM for the treatment of some diseases is very promising, especially in ulcerative colitis, tumor-related diseases, and some liver diseases. This study provides a more comprehensive analysis of the current status and significant areas of research in the field of macrophage research in TCM, offering valuable insights for prospective research endeavors.

**Conclusion:**

Macrophage-related studies in TCM have garnered increasing attention from global scholars from researchers worldwide, and are expected to become a hotspot for targeting macrophages to develop new drugs to treat diseases in the future. This study comprehensively analyzes the current status and hotspots of macrophages in Chinese medicine, which can provide valuable references for future research.

## 1 Introduction

In the late 19th century, Elie Metchnikoff introduced the concept of the “macrophage,” thus initiating the study of innate immunity (1). In addition to phagocytosis, macrophages have been shown to encode a variety of pattern recognition receptors (PRRs). These receptors have been identified as being critical for sensing pathogens and tissue damage (2–5). Macrophages serve dual roles as anti-pathogen effectors and as immune regulators. In this capacity, they release antimicrobial peptides or kill cells to limit pathogens. Furthermore, macrophages have been shown to initiate and coordinate both local and systemic immune activation processes by secreting cytokines, chemokines, and growth factors, as well as presenting antigens to adaptive immune cells (6). Macrophages are found in almost every tissue in the body, including tissue-resident alveolar macrophages, peritoneal macrophages, and Kupffer cells in the liver. These macrophages sense both exogenous pathogens and pathogens from the gastrointestinal tract. Additionally, bone marrow-derived monocytes accelerate their differentiation into macrophages upon sensing pathogen-associated molecular patterns (PAMPs) or damage-associated molecular patterns (DAMPs), enabling them to better respond to pathogens and injury (7). Beyond their role in responding to pathogens and injury, macrophages have been shown to exhibit critical regulatory activity at various stages in the process of tissue repair and regeneration (8, 9). The multifaceted functions of macrophages in innate immunity, pathogen response, and tissue homeostasis make them a compelling subject for study in the context of disease treatment and management.

The therapeutic and preventive efficacy of TCM in disease management has been increasingly validated by researchers. For instance, Yi-Yi-Fu-Zi-Bai-Jiang-San (YYFZBJS), along with its constituent herbs including Yi-yi-ren (Semen Coicis), Fu-Zi (monkshood), and Bai-jiang-cao (Herba Patriniae), has been demonstrated to exhibit diverse pharmacological activities, such as anti-cancer properties (10–12). Studies utilizing both animal models and clinical trials have revealed that YYFZBJS can ameliorate the progression of colorectal cancer by modulating immune cell regulation mediated through the gut microbiota (13). Recent studies have increasingly focused on the role of TCM in ameliorating various diseases through the modulation of macrophage function. For instance, macrophages are recognized as pivotal immune cells in the pathogenesis of atherosclerosis (14). Investigations into the therapeutic mechanisms of the clinically approved Qing-Xue-Xiao-Zhi formula (QXXZF) for atherosclerosis treatment have revealed that QXXZF alleviates atherosclerotic progression by s by inhibiting macrophage lipid accumulation and inflammatory response (15). As the most abundant immune cell population within the tumor microenvironment (TME), macrophages play a pivotal role in recognizing and phagocytosing tumor cells through nonspecific immune responses, making macrophage-targeted therapy a focus of increasing attention among researchers (16). Clinical evidence has demonstrated the promising therapeutic efficacy of the traditional Chinese medicine formulation Danzhi Xiaoyao Powder (DZXY) in breast cancer treatment. Mechanistic studies have revealed that DZXY enhances the phagocytic capacity of macrophages against tumor cells by disrupting ALOX15/PEBP1 interaction and inhibiting phospholipid peroxidation (17).

TCM has made substantial contributions to the field of disease treatment, particularly within the context of complementary and alternative therapies. Despite its limitations and deficiencies, the study of TCM warrants serious consideration (18). However, the precise mechanisms of action of TCM in diseases treatment remain unclear due to the complex composition of ingredients and multi-targeted therapeutic features. A substantial body of evidence indicates that immune mechanisms are implicated in a wide range of diseases. TCM has been demonstrated to exert pharmacological effects on the immune system, with numerous studies focusing on its immunomodulatory properties (19). There is growing evidence suggesting that TCM can influence immune cells and certain cytokines associated with immune responses (20, 21). Furthermore, TCM has the capacity to modulate the activity of both the innate immune system and the cellular subpopulations of adaptive immunity (19, 22). Given the significant roles of both TCM and macrophages in immunity, there has been an increasing number of studies investigating the effects of macrophages on TCM in recent years.

The term “bibliometric” refers to the application of statistical methods to analyze published literature and demonstrate relationships within a body of work. The concept was initially introduced by Pritchard in 1969, who defined it as “the application of mathematical and statistical methods to books and other media of communication” with the objective of illuminating the processes of written communication and the development of the discipline in which it is expressed (23). Bibliometric analysis is a widely employed research method that utilizes quantitative data to evaluate the research output of countries, journals, institutions, authors, and publications within a specific field. Additionally, it can be utilized to examine the frequency and distribution of references and keywords in published works (24). Furthermore, bibliometric analysis offers a visual representation of the collaborative relationships between countries, authors, and institutions, which can assist researchers in rapidly comprehending the characteristics of research in a given field. This information can also provide direction and guidance for future research endeavors (25).

A series of bibliometric analyses have been carried out to investigate patterns and frontiers in the domain of immunotherapy (26, 27). Similarly, bibliometric studies have been performed on literature related to macrophages (28, 29). Nevertheless, there is a paucity of bibliometric research on TCM, despite the existence of a few studies in this area. Of particular note, to the best of our knowledge, no bibliometric analysis has been conducted specifically on the literature related to macrophages in the field of TCM. This gap in the literature highlights the need for a comprehensive bibliometric examination of research at the intersection of macrophages and TCM. To address this gap, we employed bibliometric methods to analyze the literature pertinent to macrophages in the domain of TCM.

The sudden outbreak of COVID-19, in particular, has led to the extensive involvement of TCM in its treatment, demonstrating remarkable therapeutic efficacy (30, 31). The integration of TCM has significantly reduced mortality rates even in the absence of specific antiviral drugs (32). This notable achievement has sparked global interest in the application of TCM across various therapeutic domains. However, the surge in interest in TCM research in the new century actually originated from its therapeutic effects against another respiratory coronavirus, SARS-CoV (33, 34). The pivotal year of 2003 marks this time point as a critical starting point for our research. Our current bibliometric study, conducted in 2024, incorporates literature up to 2023 as the most recent inclusion criterion for our comprehensive search strategy, thereby establishing a systematic review period from 2003 to 2023.

## 2 Materials and methods

### 2.1 Database and search strategy

**Figure 1:**
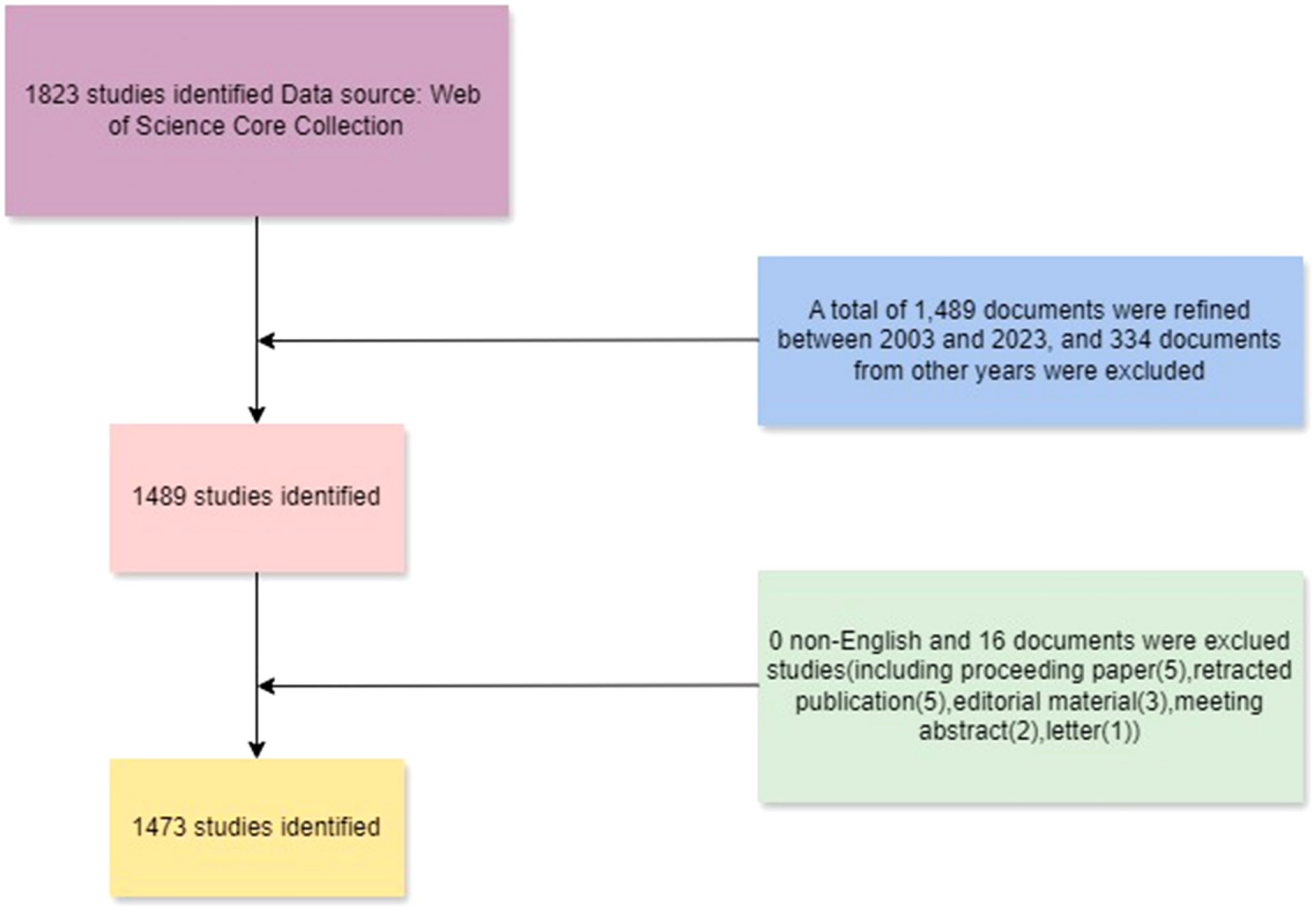
Flowchart of Literature selection and inclusion process.

Literature for the bibliometric analysis was selected from the Web of Science Core Collection, spanning from January 1, 2003, to December 31, 2023. To minimize deviations due to the rapid updating of the database, all literature searches were conducted within a single day (November 23, 2024). The following search terms were used: (TS= (“Macrophage” OR “Macrophages”) AND TS= (“Traditional Chinese medicine” OR “Chinese herbal medicine” OR “Chinese herbology” OR “Chinese medicine” OR “Chinese herb” OR “Chinese patent medicine” OR “Chinese herbal preparation” OR “Chinese herbal decoction”)). The initial search yielded a total of 1,489 documents. For the purpose of this study, only articles and reviews written in English were considered. After applying these inclusion criteria, a total of 1,473 documents were ultimately included in the analysis, comprising 1,366 articles and 107 reviews. The detailed screening process is illustrated in **Figure 1**.

### 2.2 Data analysis

The software programs Microsoft Excel (version 2021MSO) and Microsoft Word (version 2021MSO) were used for the creation of tables and the writing of the manuscript, respectively. For the visual analysis and data plotting, several specialized tools were employed, including the bibliometrix package (version 4.1, https://www.bibliometrix.org) of R software (version 4.0), VOSviewer (version 1.6.20), and CiteSpace (version 6.2.R4). To ensure the accuracy and reliability of the data, two authors independently performed the data extraction and analysis processes. This dual-review approach helps to minimize errors and biases, enhancing the overall quality and trustworthiness of the study results.

## 3 Results

### 3.1 An overview of publications on macrophages in TCM

Employing the aforementioned search strategy, a total of 1,473 papers published between 2003 and 2023 were retrieved from the Web of Science Core Collection. The bibliometric analysis revealed that these publications had collectively received a total of 39,315 citations, with an average of 26.69 citations per article. Furthermore, the H-index for the entire body of retrieved publications was calculated to be 79.

### 3.2 The annual trend in the number of publications

**Figure 2:**
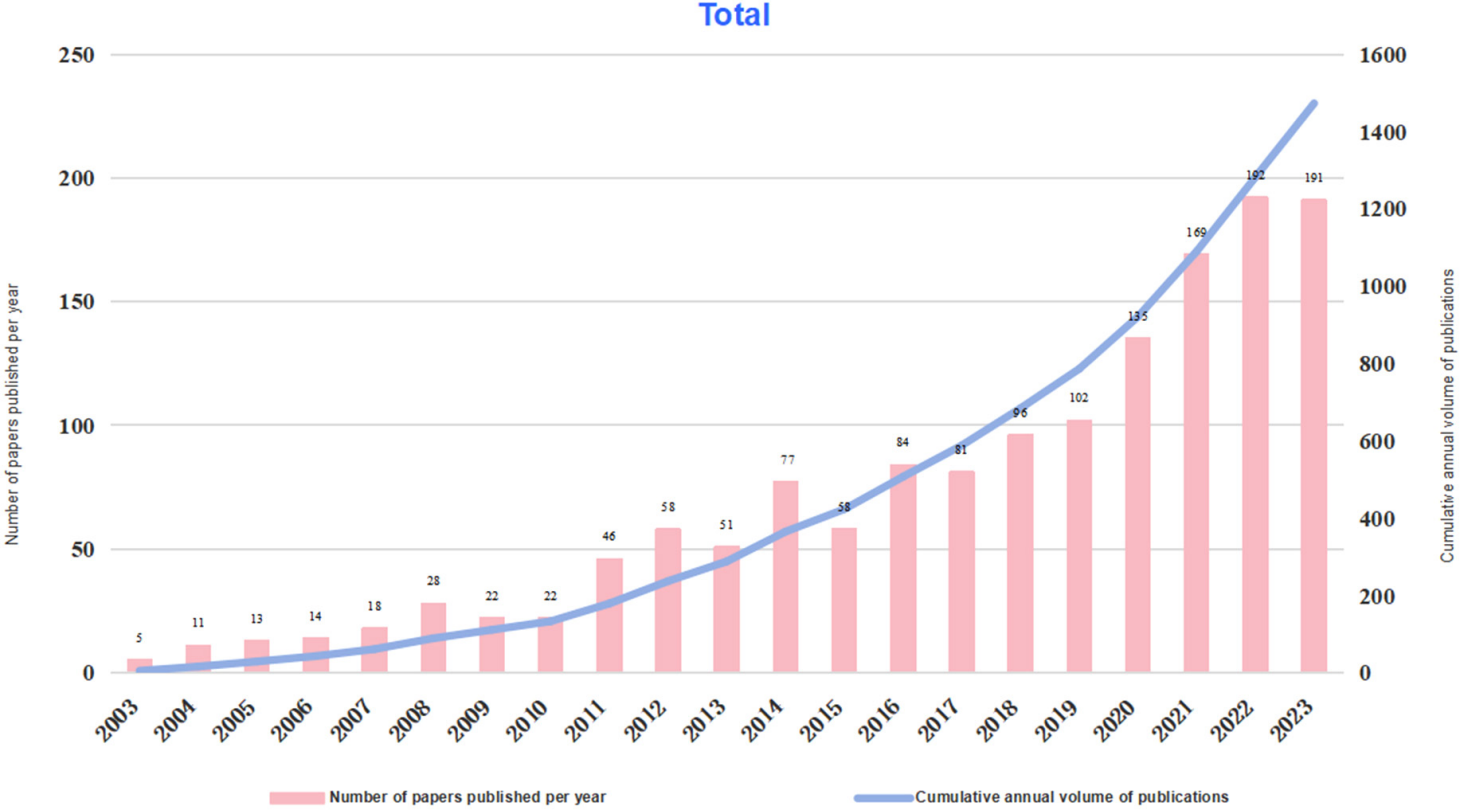
Overview plot of annual publications and cumulative annual publications of macrophages in Traditional Chinese Medicine from 2003 to 2023.

**Figure 2** illustrates the annual and cumulative number of publications on macrophage-related research in TCM from 2003 to 2023. Although the annual publication counts exhibited fluctuations, it demonstrated an overall upward trend starting from 2003, reaching a peak of 192 articles in 2022. Concurrently, the cumulative number of publications increased steadily each year, culminating in a total of 1,473 articles by 2023. These data underscore the growing interest and sustained focus on macrophage research within the field of TCM.

### 3.3 Analysis of the contributions of countries/regions

**Table 1** presents the ten countries/regions with the highest number of publications on macrophages in TCM. China emerged as the most prolific contributor, with a total of 1,336 papers, including 1,201 from Mainland China and 135 from Taiwan, China. The United States (88) and South Korea (74) followed as the second and third most productive countries, respectively. In addition to the number of publications, Mainland China also demonstrated the highest research impact, with an H-index of 71 and a total of 30,623 citations. However, when considering the average number of citations per paper, Mainland China (25.5) ranked higher only than Japan (16.91) among the top ten countries. Obviously, China had the highest number of citations (30623) in the field of macrophage research in TCM. Interestingly, although the United States and South Korea had a similar number of publications, the United States exhibited a significantly higher citation impact. The total number of citations for papers from the United States (4,402) was more than twice that of South Korea (1,908), and the average number of citations per paper for the United States (50.02) was nearly double that of South Korea (25.78). Furthermore, the United States had a higher H-index (37) compared to South Korea (26), indicating a greater number of highly cited papers from the United States. These findings highlight the substantial contributions of Chinese researchers to the field of macrophage research in TCM, both in terms of publication output and overall research impact. However, the relatively lower average citation count for papers from Mainland China suggests potential differences in the international visibility and influence of the research compared to countries like the United States.

**Table 1:** Top ten countries/regions with the highest number of publications.

Rank	Countries/Regions	Count	% of (1655)	Citations	Average Citations	H-index
1	CHINA	1201	72.56797583	30623	25.5	71
2	CHINA TAIWAN	135	8.157099698	4131	30.6	37
3	UNITED STATES	88	5.317220544	4402	50.02	37
4	SOUTH KOREA	74	4.471299094	1908	25.78	26
5	JAPAN	34	2.054380665	575	16.91	14
6	AUSTRALIA	26	1.570996979	1255	48.27	18
7	ENGLAND	9	0.543806647	314	34.89	7
8	AUSTRIA	8	0.483383686	305	38.13	8
9	INDIA	7	0.422960725	265	37.86	7
10	GERMANY	5	0.302114804	224	44.8	5
10	THAILAND	5	0.302114804	234	46.8	4

**Figure 3:**
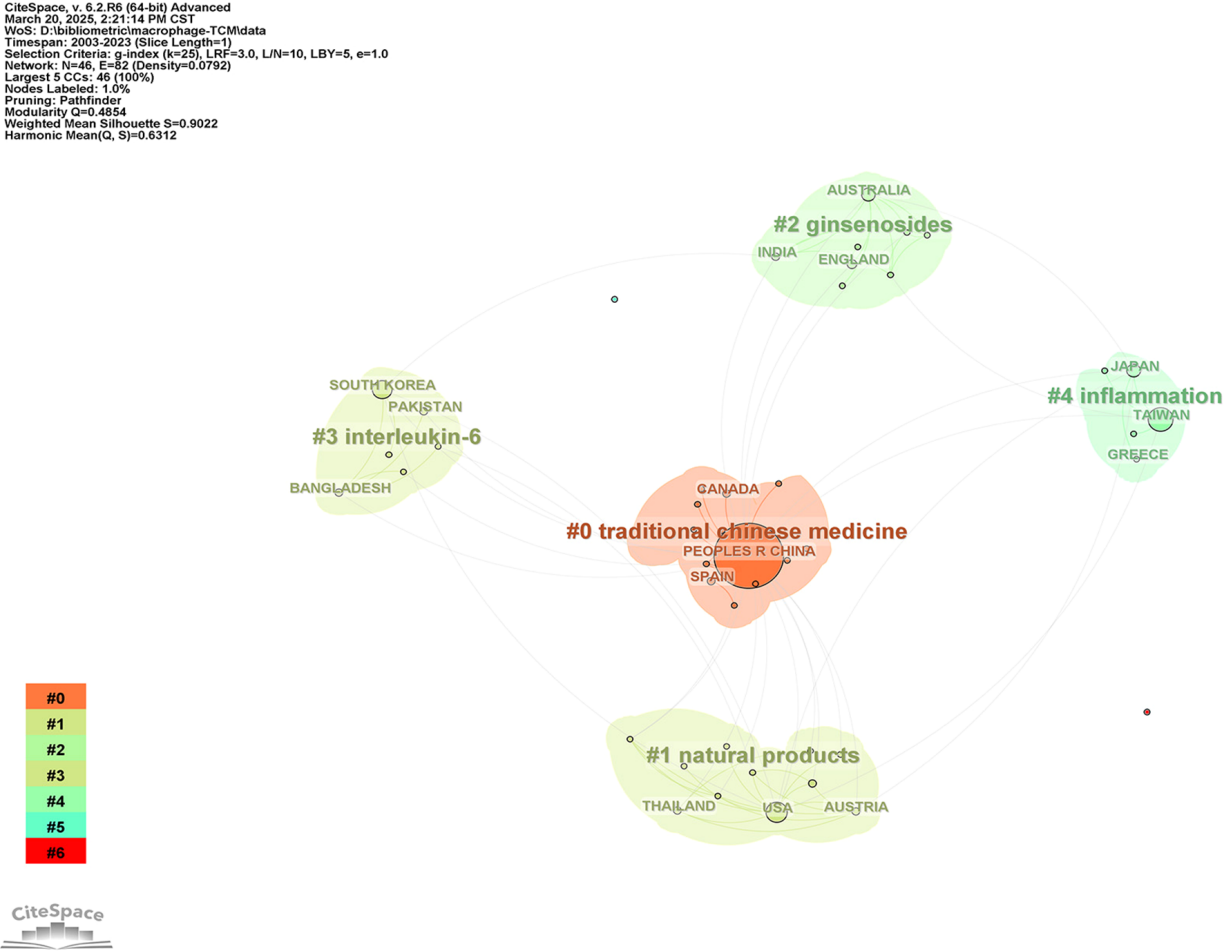
Co-occurrence analysis between countries/regions based on CiteSpace.

In order to explore the collaborative relationships between countries/regions, we used CiteSpace6.2R4 to analyze the co-occurring relationships between countries/regions and performed clustering, and obtained a countries/regions cluster map(**Figure 3**). The Modularity Q value of a cluster typically ranges from 0 to 1, with higher values indicating better clustering performance. Generally, a Q value greater than 0.3 suggests that the network exhibits a significant cluster structure. On the other hand, the Weighted Mean Silhouette S value ranges from -1 to 1, where values closer to 1 indicate high internal consistency within clusters and clear separation between clusters. Our clustering results yielded Q = 0.4854 and S = 0.9022, demonstrating that the identified clusters not only possess a significant modular structure but also exhibit strong internal consistency and distinct separation. **Figure 3** shows the formation of collaborative networks between countries/regions engaged in research pertaining to macrophages in Chinese medicine, with China exhibiting the highest centrality(1.38). From the **Figure 3**, We find that China is in Cluster #0(traditional chinese medicine) with Spain and Canada; China Taiwan, Japan, and Greece are in Cluster #4(inflammation); the United States, Austria, and Thailand are in Cluster #1(natural products); India, Australia, and England are in Cluster #2(ginsenosides); and Pakistan, South Korea, and Bangladesh are in Cluster #3(interleukin-6). Countries/Regions in the same cluster tend to have similar research directions, which means that countries/regions in the same cluster are more likely to co-operate with each other.

### 3.4 Analysis of the contributions by affiliations

**Figure 4:**
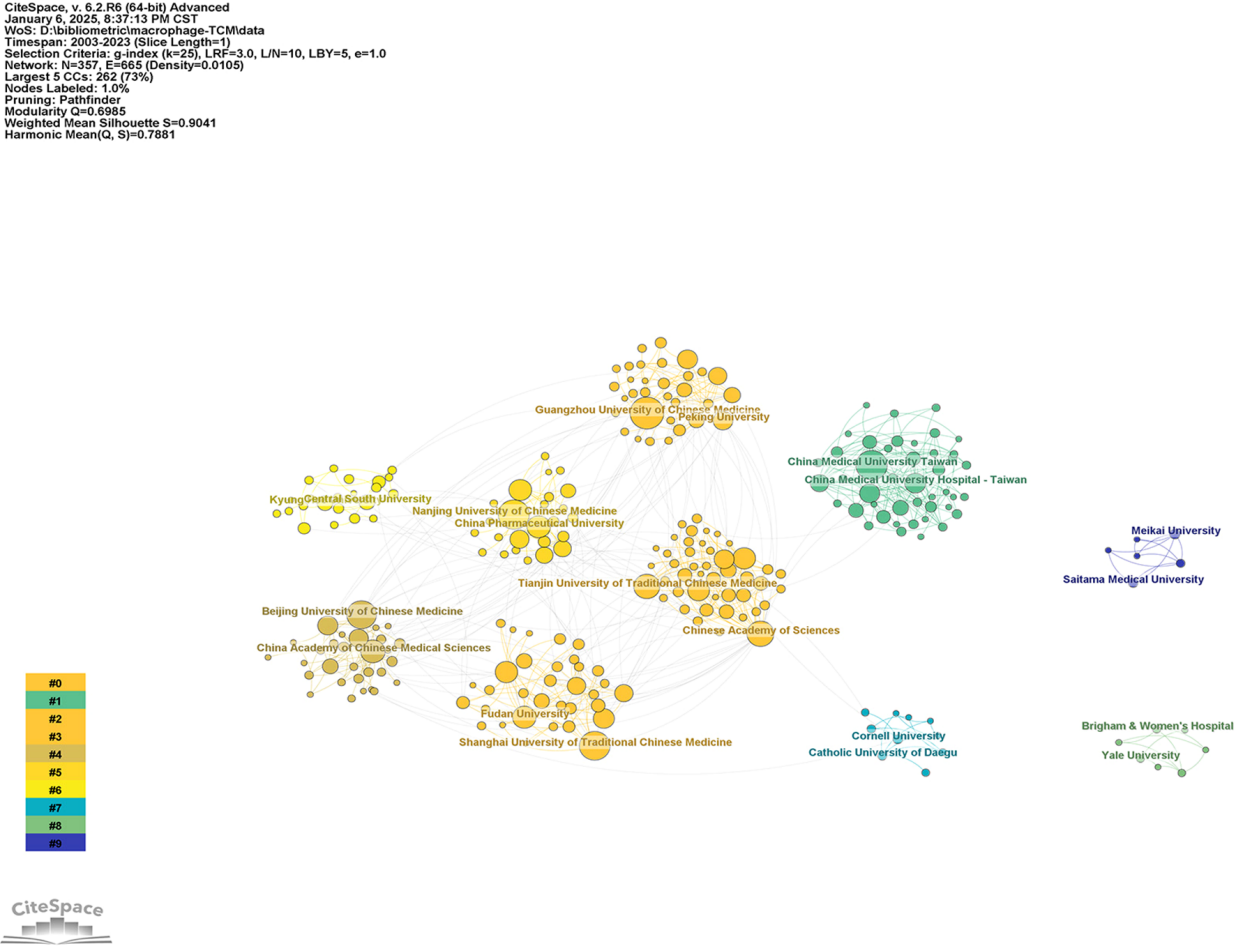
Co-occurrence analysis of issuing institutions related to macrophages in Traditional Chinese Medicine research.

**Table 2** presents the top 10 institutions ranked by publication volume in macrophage-related research within TCM. All leading institutions are based in China, with Guangzhou University of Chinese Medicine demonstrating the highest publication output (76 articles), followed by Nanjing University of Chinese Medicine (66 articles). Notably, China Medical University Taiwan and Shanghai University of Traditional Chinese Medicine share the third position with 64 publications each; however, China Medical University Taiwan exhibits superior performance in citation metrics, including total citations, average citations per paper, and H-index. To analyze institutional collaboration patterns, we employed CiteSpace6.2R4 to generate a co-occurrence network visualization (**Figure 4**). The resulting network comprises 357 nodes and 665 connections, with a network density of 0.0105. The clustering analysis yielded a Modularity Q value of 0.6985 and a Weighted Mean Silhouette S value of 0.9041, indicating that the identified clusters are both statistically significant and highly robust in terms of internal consistency and inter-cluster separation. Within this network, the Chinese Academy of Sciences emerges as the most influential institution, positioned in Cluster #0, with the highest centrality (0.41) and degree (35) metrics. These quantitative indicators suggest that the Chinese Academy of Sciences plays a pivotal role in macrophage research within TCM.

**Table 2:** Top 10 affiliations with the highest number of publications.

Rank	Affiliations	Countries/Regions	Count	% of (2346)	Citations	Average Citations	H-index
1	GUANGZHOU UNIVERSITY OF CHINESE MEDICINE	CHINA	76	3.239556692	2000	26.32	26
2	NANJING UNIVERSITY OF CHINESE MEDICINE	CHINA	66	2.813299233	1360	20.61	22
3	CHINA MEDICAL UNIVERSITY TAIWAN	CHINA TAIWAN	64	2.728047741	1969	30.77	26
3	SHANGHAI UNIVERSITY OF TRADITIONAL CHINESE MEDICINE	CHINA	64	2.728047741	1236	19.31	24
5	BEIJING UNIVERSITY OF CHINESE MEDICINE	CHINA	56	2.387041773	961	17.16	18
6	CHINESE ACADEMY OF SCIENCES	CHINA	48	2.046035806	1279	26.65	21
7	TIANJIN UNIVERSITY OF TRADITIONAL CHINESE MEDICINE	CHINA	47	2.00341006	943	20.06	20
8	CHINA ACADEMY OF CHINESE MEDICAL SCIENCES	CHINA	39	1.662404092	1112	28.51	20
9	FUDAN UNIVERSITY	CHINA	38	1.619778346	750	19.74	16
10	SHANGHAI JIAO TONG UNIVERSITY	CHINA	37	1.5771526	1115	30.14	21

### 3.5 Analysis of authors

**Figure 5:**
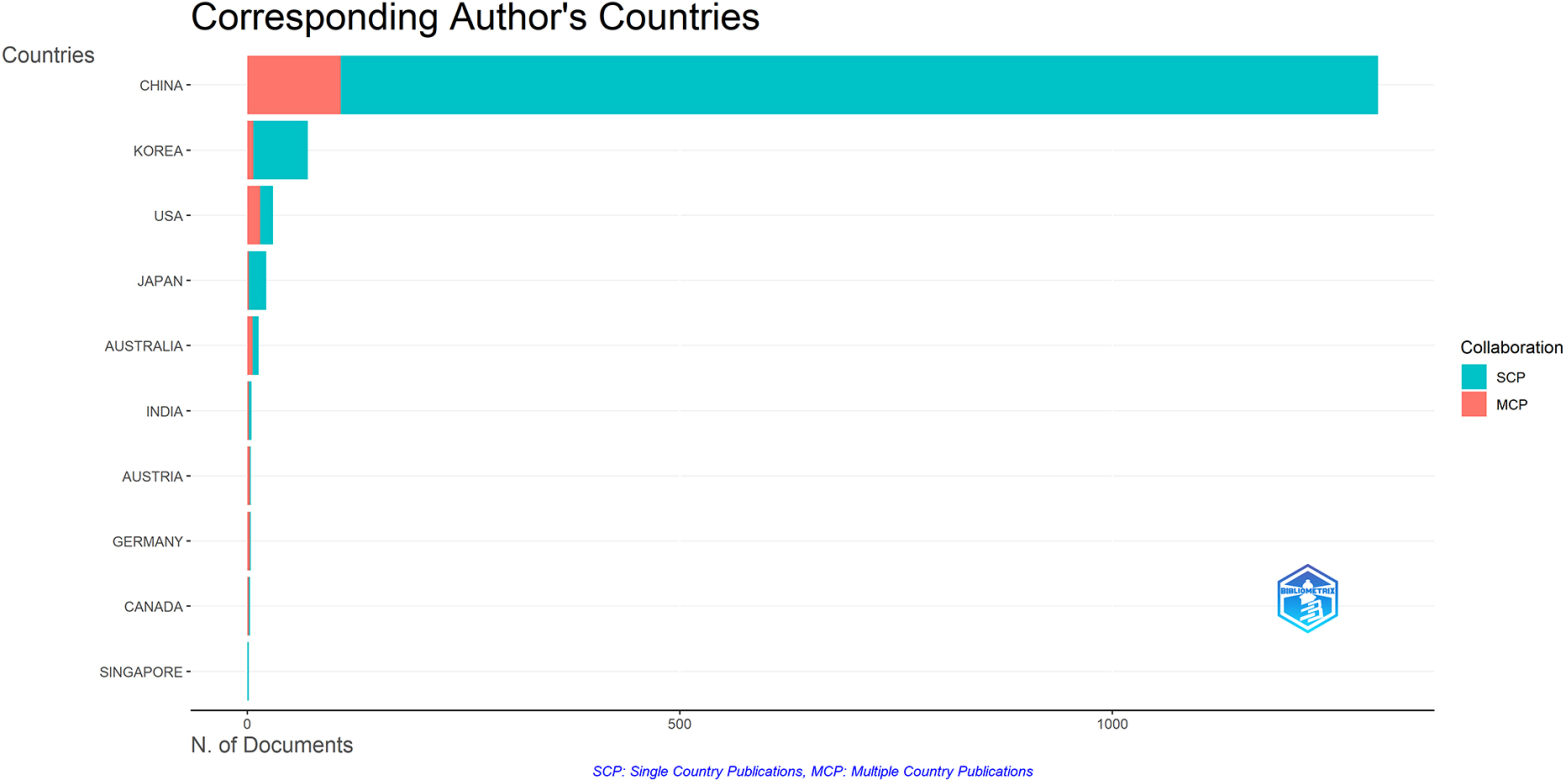
MCP and SCP in the top 10 countries in terms of number of publications by corresponding authors.

**Table 3** presents the top ten authors ranked by publication output. All leading authors are affiliated with Chinese institutions, with Leung, Ping-Chung from the Chinese University of Hong Kong demonstrating the highest productivity (12 publications), accumulating 532 citations and achieving an H-index of 11. Notably, Fung, Kwok Pui, also from the Chinese University of Hong Kong, exhibits the highest citation impact with an average of 49.1 citations per publication. Analysis of corresponding author’s Countries/Regions, visualized using the bibliometrix package in R (**Figure 5**), reveals the distribution of Multi-Country Publications (MCP) and Single-Country Publications (SCP) across countries/regions. While China maintains the highest absolute values in both MCP and SCP metrics, its international collaboration ratio (MCP/total publications) is 8.3%, significantly lower than that of countries ranked second through ninth. This relatively low international collaboration rate suggests a predominant tendency toward domestic collaboration among Chinese authors in this field. Although this pattern may indicate strong domestic research networks, expanding international collaborative endeavors could potentially enhance the global impact and diversity of research perspectives in this domain.

**Table 3:** Top 10 authors with the highest number of publications.

Rank	Authors	Countries/Regions	Affiliations	Count	Citations	Average Citations	H-index
1	Leung, Ping-Chung	CHINA	The Chinese University of Hong Kong	12	532	44.33	11
2	ZHOU, LIANG	CHINA	Guangzhou Brain Hospital	11	316	28.73	9
3	Dong, Tina T. X.	CHINA	Hong Kong University of Science & Technology	10	303	30.3	8
3	Lau, Clara Bik-San	CHINA	University of Hong Kong	10	473	47.3	9
3	Lu, Zi-Bin	CHINA	Southern Medical University	10	216	21.6	9
3	Huang, Guan-Jhong	CHINA TAIWAN	China Medical University Taiwan	10	434	43.4	9
3	Fung, Kwok Pui	CHINA	The Chinese University of Hong Kong	10	491	49.1	9
8	Duan, Jin-Ao	CHINA	Nanjing University of Chinese Medicine	9	282	31.33	9
8	Cao, Hui-Hui	CHINA	Shaanxi University of Science & Technology	9	207	23	9
8	Liu, Jun-Shan	CHINA	Southern Medical University - China	9	215	23.89	9

### 3.6 Analysis of the contributions of journals

**Figure 6:**
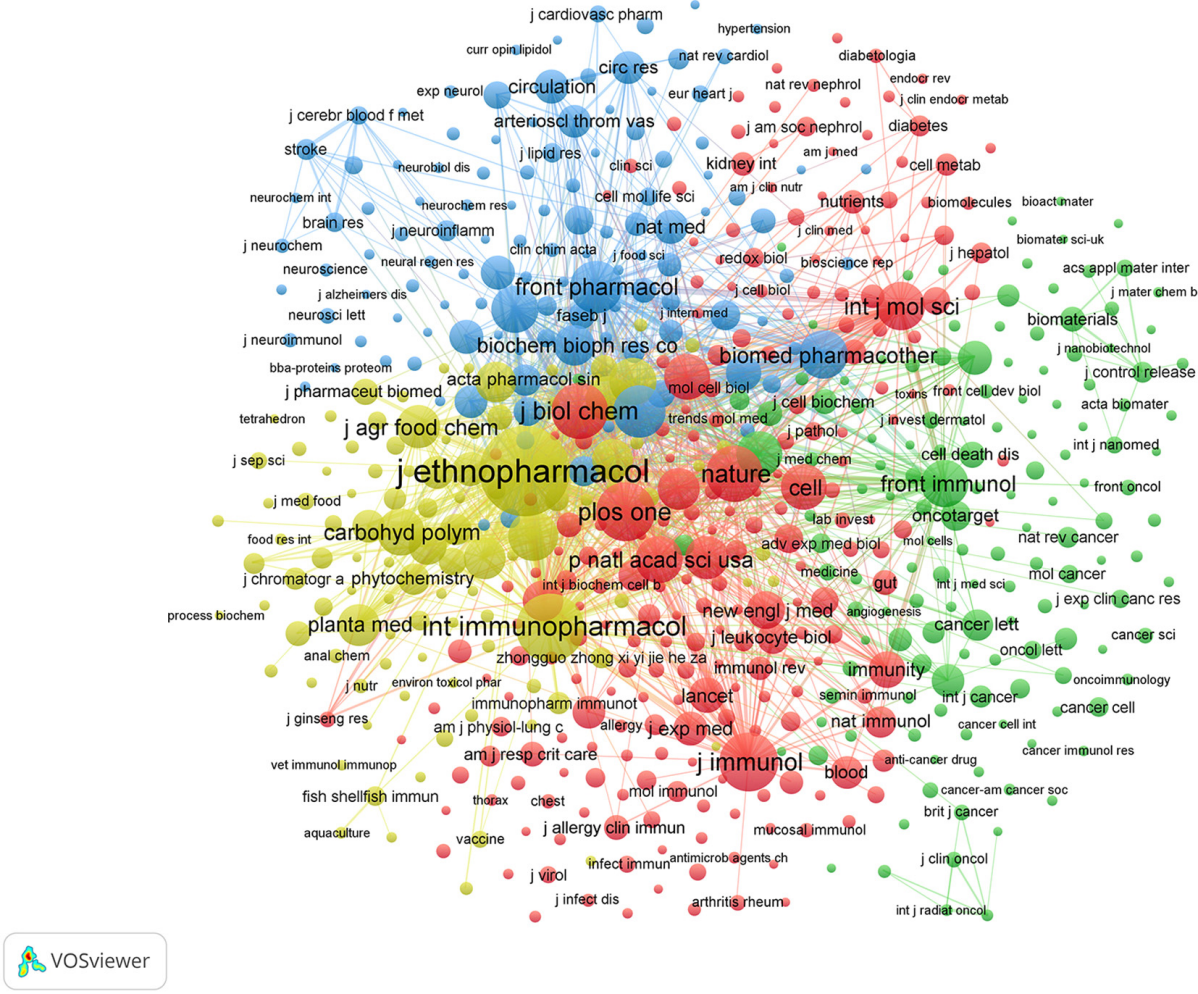
Co-citation network of journals.

In network visualizations, node size is indicative of citation frequency, while link width represents the connection strength between nodes. A total corpus of 7,191 journals in the field was selected, and 595 documents were chosen using a minimum threshold of 20 citations. **Table 4** presents the top 10 journals ranked by citation metrics. Among the 595 cited journals, Journal of Ethnopharmacology, International Immunopharmacology, and Frontiers in Pharmacology emerged as the most influential publications based on citation metrics. The application of co-citation analysis has yielded the identification of four distinguished journal clusters (**Figure 6**). The primary cluster (red, 229 items) encompasses high-impact generalist journals, including Nature, Cell, Science, and PLOS One, as well as prominent immunology journals such as Journal of Immunology and Immunity. The second cluster (green, 143 items) comprises both multidisciplinary journals, such as Scientific Reports and Nature Communications, and specialized oncology publications, including Oncotarget, Cancer Research, and Cell Death and Disease. The third cluster (blue, 118 items) consists predominantly of pharmacology-focused journals, including Biomedicine & Pharmacotherapy, Frontiers in Pharmacology, and European Journal of Pharmacology. The fourth cluster (yellow, 105 items) is characterized by journals specializing in phytomedicine and natural products, such as Journal of Ethnopharmacology, International Journal of Biological Macromolecules, Journal of Agricultural and Food Chemistry, and Phytomedicine.

**Table 4:** Top ten most cited journals.

Rank	Source	Citations	Total link strength
1	Journal of Ethnopharmacology	1921	103493
2	International Immunopharmacology	1079	58528
3	PLOS One	832	42864
4	Journal of Immunology	822	35358
5	Nature	741	37377
6	Frontiers in Pharmacology	637	50636
7	Evidence-based Complementary and Alternative Medicine	636	39315
8	International Journal of Molecular Sciences	569	40574
9	Biomedicine and Pharmacotherapy	554	41055
10	Frontiers in Immunology	543	36379

### 3.7 Keywords analysis

#### 3.7.1 Keywords co-occurrence analysis

**Figure 7:**
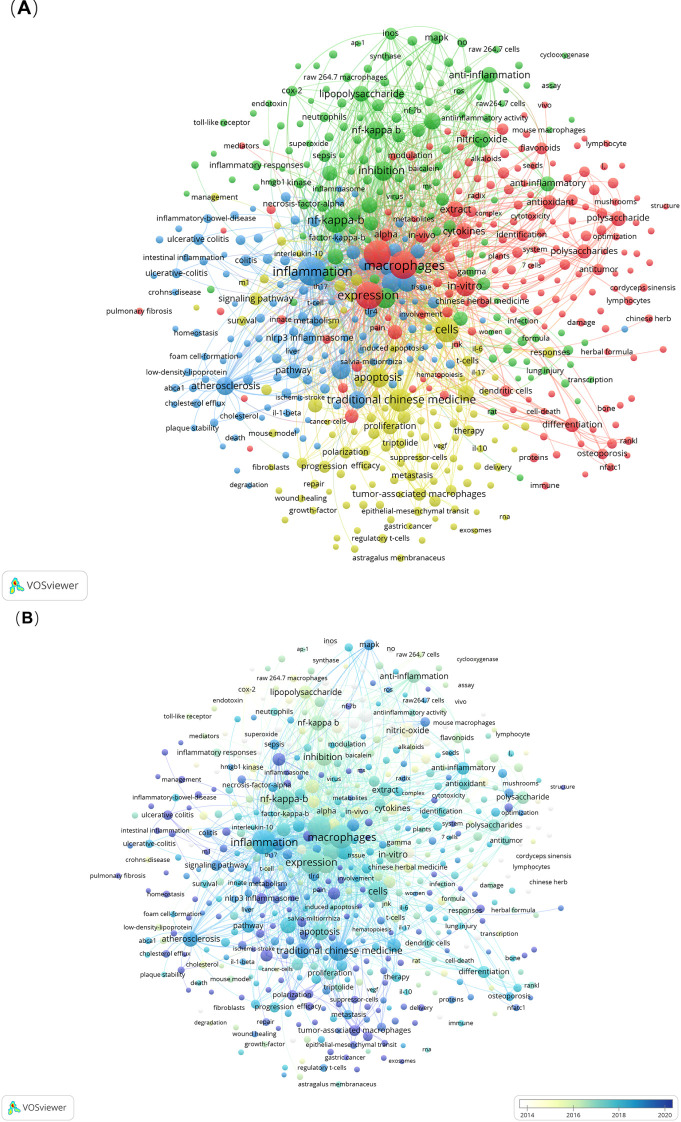
Network visualization of keyword co-occurrence. **(A)** Keyword cluster map based on different research field. **(B)** Overlay visualization based on keyword co-occurrence network.

We used VOSviewer for visual co-occurrence analysis of keywords. A total of 6363 keywords in the field published articles, and 546 documents were screened using a threshold of a minimum of 5 total occurrences. The analysis yielded “macrophages” and “inflammation” as the predominant keywords, thereby indicating the central focus of TCM research on the function of macrophages in inflammatory processes. Notably, pathway analyses have concentrated significantly on the NF-κB signaling cascade, a canonical inflammatory pathway. The co-occurrence network visualization delineates four distinct clusters (**Figure 7A**). The primary cluster (red, 168 items) encompasses terms associated with *in vitro* macrophage studies in TCM, including “macrophage” “expression” “cytokine” “*in vitro*” and “differentiation”. The second cluster (green, 143 items) focuses on NF-κB pathway regulation, featuring keywords such as “NF-κB” “inhibition” and “induction”. The third cluster (blue, 124 items) centers on inflammation-related pathways and specific pathological conditions, incorporating both molecular mediators (Nrf2, NLRP3, HMGB1) and disease states (atherosclerosis, ulcerative colitis, liver diseases). The fourth cluster (yellow, 111 items) emphasizes cancer biology, cell death mechanisms, and macrophage polarization, with keywords including “TCM” “apoptosis” and “cancer” demonstrating a shift from inflammatory processes toward oncological applications. Temporal analysis of keyword occurrence, visualized through overlay mapping (**Figure 7B**), reveals the chronological evolution of research themes. The visualization employs a color gradient from white (earlier occurrences) to blue (recent occurrences). This temporal analysis indicates that keywords within the red and green clusters emerged earlier in the literature, while those in the blue and yellow clusters represent more recent research directions.

#### 3.7.2 Trend and emergence analysis of keywords

**Figure 8:**
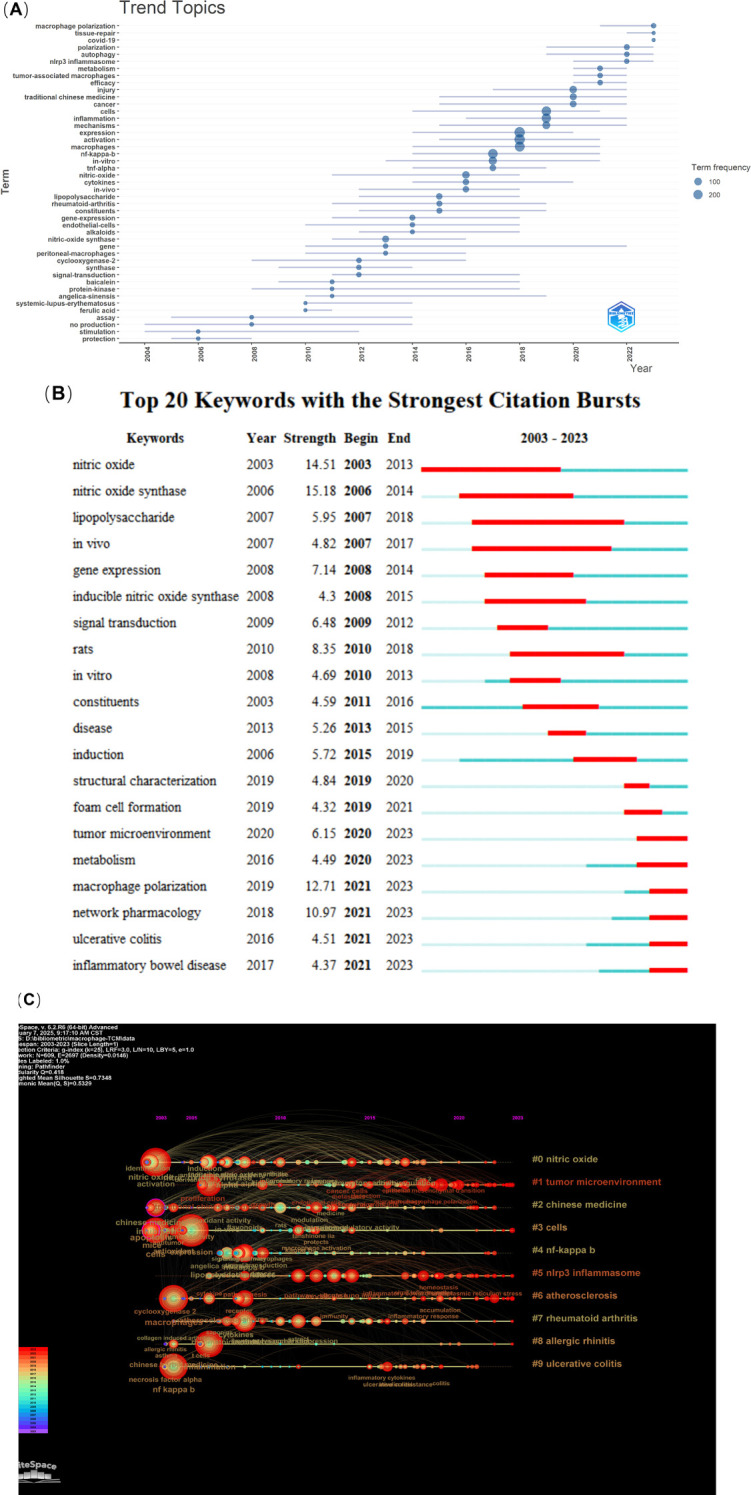
The phenomenon of keyword emergence and the subsequent analysis of trends. **(A)** Trend topics on macrophages in TCM. **(B)** Emergent map of keywords. **(C)** Keyword timeline.

In order to identify the trends of macrophages in the field of TCM, we plotted the graph in **Figure 8A** using the bibliometrics package of the R software. Prior to 2020, the subject matter predominantly encompassed the fields of inflammation, mechanisms, expression, and the NF-κB pathway. However, following this period, there was a notable shift in focus toward metabolism, tumor-associated macrophages, NLRP3 inflammasome, and, subsequently, autophagy, tissue-repair, and polarization. The advent of the global pandemic of the novel coronavirus disease (Covid-19) precipitated an inevitable surge in research activity within this field. Notwithstanding, the topic of macrophage polarization has emerged as the most discussed area of investigation in the context of TCM.

Keywords exhibiting citation bursts are those that have been the subject of frequent discussion within a given field over a defined period of time. Burst word analysis can reveal the most popular research in a research field during a specific period. The top 20 keywords with the strongest citation bursts are shown in **Figure 8B**. An initial bibliometric analysis identified two predominant research emphases: nitric oxide (2003-2013) and nitric oxide synthase (2006-2014), with burst strengths of 14.51 and 15.18, respectively. This research emphasis likely correlates with the 1998 Nobel Prize in Medicine/Physiology, awarded to Furchgott, Ignarro, and Murad for their discovery of nitric oxide as a signaling molecule in the cardiovascular system. Prior to 2013, prominent research keywords included lipopolysaccharide, *in vivo*, *in vitro*, rat models, gene expression, and signal transduction. This indicates that researchers primarily concentrated on model development and fundamental experimental investigations during this period. It is noteworthy that while nitric oxide synthase continued to be a prominent subject in research, the focus underwent a shift toward inducible nitric oxide synthase. The year 2013 marked a transition from *in vivo* and *in vitro* experimental paradigms toward disease-oriented research. New research terms emerged, including structural characterization, tumor microenvironment, metabolism, and foam cell formation, albeit with relatively modest burst strengths. The period between 2021 and 2023 witnessed two major research trends: macrophage polarization and network pharmacology, demonstrating burst strengths of 12.71 and 10.97, respectively. Concurrently, ulcerative colitis and inflammatory bowel disease emerged as the predominant disease-specific research focuses in the field.

The temporal distribution and relationships among clusters were visualized through timeline analysis, as illustrated in **Figure 8C**. The visualization reveals that clusters #0 (nitric oxide), #2 (chinese medicine), #4 (nf-kappa b), and #9 (ulcerative colitis) have reached saturation in their research trajectories. Conversely, clusters #1 (tumor microenvironment), #3 (cells), #5 (nlrp3 inflammasome), #6 (atherosclerosis), #7 (rheumatoid arthritis), and #8 (allergic rhinitis) exhibit sustained research activity extending to the present, indicating their status as persistent areas of scientific investigation. These latter clusters represent enduring research priorities within the field.

## 4 Discussion

### 4.1 Overview of the study

The bibliometric analysis of literature from 2003 to 2023 examining macrophages in TCM research revealed a consistent upward trajectory in publication frequency. A notable acceleration in publication rate was observed post-2019, potentially correlating with TCM’s significant role in Covid-19 prevention and treatment protocols. This quantitative analysis demonstrates the increasing prominence of macrophage-focused investigations within TCM research frameworks. The bibliometric analysis reveals that China dominates the research landscape with 1,336 publications, while Guangzhou University of Chinese Medicine emerges as the most productive institution. The highest individual contribution comes from Leung, Ping-Chung, further solidifying China’s preeminence in TCM research. Notably, countries with historical Chinese cultural influence, including South Korea, Japan, and Thailand, also demonstrate substantial research output in this field. This geographical distribution of research activity reflects both the historical origins and contemporary development of TCM. The bibliometric analysis reveals an increasing international engagement in research related to macrophages in TCM, with China maintaining a predominant position. The increasing global participation and expanding collaborative networks indicate progressive internationalization of this research field. This comprehensive bibliometric evaluation provides essential insights into research trends, collaborative patterns, and knowledge structures, thereby establishing a foundation for future investigations and potential research directions.

### 4.2 Summary and analysis of hotspots

Through systematic analysis of keyword chronology, clustering patterns, and emerging topics, we identified the research hotspots and frontiers in TCM’s macrophage-related investigations. Our bibliometric evaluation has identified three principal research domains within the intersection of macrophage biology and TCM.

The primary research domain encompasses the analysis of macrophage-associated pathologies within the TCM framework. A meticulous examination of the extant literature has identified cancer, ulcerative colitis, atherosclerosis, and liver disease as the predominant research areas. Notably, cancer immunotherapy has emerged as a significant therapeutic approach, with TCM demonstrating efficacy through targeted modulation of immune cells, particularly tumor-associated macrophages (TAMs). Specific examples include the Aiduqing formula, which inhibits the expression and secretion of CXCL1 by TAMs, thereby inhibiting the chemotaxis and differentiation of initial CD4+ T cells to Tregs as a means to inhibit breast cancer metastasis (36). Additionally, Compound Bitter Ginseng Injection enhances hepatocellular carcinoma sensitivity to sorafenib through attenuation of TAMs-mediated immunosuppression via TNFR1 signaling (37). Our analysis revealed that TCM ameliorates ulcerative colitis primarily through modulation of macrophage polarization phenotypes, with efficacy demonstrated across herbal formulations, mono-components, and specific herbal combinations. Huang Qin Decoction exhibits therapeutic effects through regulation of fatty acid metabolism-mediated macrophage polarization via the FFAR4-AMPK-PPARα signaling pathway (35). Ginsenoside Rg1, a mono-component, demonstrates efficacy in solid ulcerative colitis through dual mechanisms: regulation of M1/M2 macrophage polarization and restoration of intestinal flora homeostasis (38). Furthermore, Huang Lian Gan Jiang decoction, utilizing the synergistic interaction between “hot” and “cold” properties of its constituents, attenuates inflammatory responses and restores intestinal barrier function in ulcerative colitis (39). Multiple TCM formulations demonstrate therapeutic efficacy in atherosclerosis through macrophage-targeted mechanisms. These include Qing Re Huo Xue Decoction, Qing-Xue-Xiao-Zhi formula, and Tiaogan Daozhuo formula, each exhibiting specific macrophage-mediated effects in atherosclerosis treatment (15, 40, 41). Furthermore, specific bioactive compounds isolated from traditional herbs demonstrate anti-atherosclerotic effects through macrophage-mediated mechanisms, including Catalpol, Isorhamnetin, and Tanshinone IIA (42–44). In liver disease, TCM demonstrates therapeutic efficacy through multiple macrophage-related mechanisms, including modulation of macrophage-microbiota interactions, regulation of macrophage pyroptosis, and control of macrophage polarization. These mechanisms are implicated in the treatment of diverse liver diseases, including acute liver injury, non-alcoholic fatty liver disease (NAFLD), and liver fibrosis (45–47).

The second major research domain focuses on macrophage polarization, which has emerged as a dominant research theme in recent years, as evidenced by our bibliometric analysis. Macrophages demonstrate remarkable phenotypic plasticity, exhibiting distinct polarization states in response to diverse inflammatory microenvironments and multiple regulatory factors (48, 49). Macrophages play a pivotal role in inflammatory diseases, including atherosclerosis, metabolic homeostasis, allergic diseases, autoimmune diseases, and type II diabetes (50). TCM demonstrates significant therapeutic potential in inflammatory conditions through its capacity to modulate macrophage polarization. This modulation occurs through multiple mechanisms: direct regulation of polarization states, modulation of signaling pathways, inhibition of complement cascade reactions, and metabolic regulation. For instance, Shenlian decoction enhances the M1/M2 macrophage ratio in hepatocellular carcinoma through complement cascade inhibition (51). Additionally, Qingfei oral liquid attenuates respiratory syncytial virus (RSV)-induced pulmonary inflammation by promoting fatty acid-dependent M1/M2 macrophage polarization via the Akt signaling pathway (52).

The third major research domain encompasses methodological approaches for investigating macrophage-TCM interactions. TCM, particularly its multi-component formulations, presents unique challenges for mechanistic studies due to its complex composition and multi-target nature. Network pharmacology has emerged as an innovative methodological framework that facilitates the transition from conventional “single-target, single-drug” paradigms to more comprehensive “network-target, multi-component” approaches in TCM research (53). Network pharmacology has gained widespread adoption in TCM research, with our bibliometric analysis revealing a significant increase in citations for this methodological approach, particularly post-2018. This methodology facilitates the mapping of TCM components to disease targets, enabling identification of differentially expressed genes. The analytical process involves construction of protein-protein interaction (PPI) networks, followed by Gene Ontology (GO) functional analysis and Kyoto Encyclopedia of Genes and Genomes (KEGG) pathway enrichment analysis of identified targets. This systematic approach enables comprehensive screening of relevant pathways, molecular functions, and biological processes. Multiple studies investigating macrophage-TCM interactions have successfully employed network pharmacology to elucidate potential therapeutic targets. For example, the potential target of Shaoyao decoction for the treatment of ulcerative colitis was screened by network pharmacology to be NLRP3, and the potential therapeutic pathway was MKP1/NF-κB (54). Similarly, researchers have found through network pharmacology that Xuanfei Baidu Decoction attenuates lung injury by modulating neutrophil and macrophage infiltration through the PD-1/IL17A pathway, which has been validated in *in-vivo* and *in-vitro* experiments (55).

### 4.3 Strengths and weaknesses

Our bibliometric analysis provides researchers with a comprehensive overview of this field; however, certain limitations warrant acknowledgment. The primary limitation stems from our exclusive utilization of the Web of Science Core Collection database, which may restrict the comprehensiveness of included literature. This methodological choice was justified by WOS Core Collection’s status as a premier scientific database, widely recognized for both its high-quality publications and particular suitability for bibliometric analyses (56–58). The data sources selected for this study demonstrate robust reliability. In addition, our search strategy was limited to the “macrophage” and “macrophages”, excluding macrophage-like cells such as Kupffer cells and monocytes. This approach may have resulted in incomplete coverage of macrophage-related studies. However, this exclusion criterion was intentionally implemented to avoid potential bias in our analysis, as certain macrophage-like cells are known to be tissue-specific, and their inclusion could introduce disease-specific tendencies that might compromise the objectivity of our findings. Furthermore, the present analysis exclusively encompasses English language literature, whereas literature in other languages has not been subjected to bibliometric analysis. This methodological approach may give rise to selection bias by excluding valuable scholarship from non-English sources. Given the widespread use of English in academic fields, which makes this language more standardized in academic applications, it is reasonable for us to choose English literature for analysis.

## 5 Conclusion

Bibliometric analyses reveal a rapid expansion in research concerning macrophages within the context of TCM. China has established itself as the leading contributor to this field’s literature, with significant contributions also emanating from Korea, the United States, and Japan. Among institutional contributors, the Guangzhou University of Chinese Medicine has demonstrated particular prominence in publication output. Recent trends indicate a notable shift in research focus toward macrophage polarization and network pharmacology approaches. Additionally, TCM studies investigating macrophage-mediated pathologies have predominantly concentrated on four key areas: ulcerative colitis, atherosclerosis, cancer and liver diseases.

## Data Availability

The original contributions presented in the study are included in the article/supplementary material. Further inquiries can be directed to the corresponding authors.

## References

[1] Cooper MD Alder MN. The evolution of adaptive immune systems. Cell. (2006) 124:815–22. doi: 10.1016/j.cell.2006.02.001 16497590

[2] Rock KL Kono H. The inflammatory response to cell death. Annu Rev Pathology: Mech Dis. (2008) 3:99–126. doi: 10.1146/annurev.pathmechdis.3.121806.151456 PMC3094097 18039143

[3] Martinon F Mayor A Tschopp J. The inflammasomes: guardians of the body. Annu Rev. (2009) 27:229–65. doi: 10.1146/annurev.immunol.021908.132715 19302040

[4] Janeway CA Jr Medzhitov R. Innate immune recognition. Annu Rev Immunol. (2002) 20:197–216. doi: 10.1146/annurev.immunol.20.083001.084359 11861602

[5] Medzhitov R Preston-Hurlburt P Janeway CA. A human homologue of the Drosophila Toll protein signals activation of adaptive immunity. Nature. (1997) 388:394–7. doi: 10.1038/41131 9237759

[6] Rivera A Siracusa MC Yap GS Gause WC. Innate cell communication kick-starts pathogen-specific immunity. Nat Immunol. (2016) 17:356–63. doi: 10.1038/ni.3375 PMC4949486 27002843

[7] Davies LC Jenkins SJ Allen JE Taylor PR. Tissue-resident macrophages. Nat Immunol. (2013) 14:986–95. doi: 10.1038/ni.2705 PMC4045180 24048120

[8] Wynn TA Vannella KM. Macrophages in tissue repair, regeneration, and fibrosis. Immunity. (2016) 44:450–62. doi: 10.1016/j.immuni.2016.02.015 PMC4794754 26982353

[9] Wynn TA Barron L. Macrophages: master regulators of inflammation and fibrosis. Semin Liver Dis. (2010) 30:245–57. doi: 10.1055/s-0030-1255354 PMC2924662 20665377

[10] Xia L Zhang B Yan Q Ruan S. Effects of saponins of patrinia villosa against invasion and metastasis in colorectal cancer cell through NF-κB signaling pathway and EMT. Biochem Biophys Res Commun. (2018) 503:2152–9. doi: 10.1016/j.bbrc.2018.08.005 30119890

[11] Asian Pacific Journal of Cancer Prevention . Available online at: https://journal.waocp.org/?sid=Entrez (Accessed March 12, 2025).

[12] Trinh TA Park SC Oh J Kim C-E Kang KS Yoo HS. Preventive effect and safety of a follicle stimulating hormone inhibitory formulation containing a mixture of coicis semen and artemisia capillaris for precocious puberty: A preliminary experimental study using female rats. Evid Based Complement Alternat Med. (2017) 2017:2906014. doi: 10.1155/2017/2906014 29348765 PMC5734006

[13] Sui H Zhang L Gu K Chai N Ji Q Zhou L. YYFZBJS ameliorates colorectal cancer progression in ApcMin/+ mice by remodeling gut microbiota and inhibiting regulatory T-cell generation. Cell Commun Signal. (2020) 18:113. doi: 10.1186/s12964-020-00596-9 32677955 PMC7367414

[14] Chistiakov DA Bobryshev YV Orekhov AN. Macrophage-mediated cholesterol handling in atherosclerosis. J Cell Mol Med. (2016) 20:17–28. doi: 10.1111/jcmm.12689 PMC4717859 26493158

[15] Li Y Zhang L Ren P Yang Y Li S Qin X. Qing-Xue-Xiao-Zhi formula attenuates atherosclerosis by inhibiting macrophage lipid accumulation and inflammatory response via TLR4/MyD88/NF-κB pathway regulation. Phytomedicine. (2021) 93:153812. doi: 10.1016/j.phymed.2021.153812 34753029

[16] Chen S Saeed AFUH Liu Q Jiang Q Xu H Xiao GG. Macrophages in immunoregulation and therapeutics. Signal Transduct Target Ther. (2023) 8:207. doi: 10.1038/s41392-023-01452-1 37211559 PMC10200802

[17] Luo X Li D-D Li Z-C Li Z-X Zou D-H Huang F. Mitigating phospholipid peroxidation of macrophages in stress-induced tumor microenvironment by natural ALOX15/PEBP1 complex inhibitors. Phytomedicine. (2024) 128:155475. doi: 10.1016/j.phymed.2024.155475 38492368

[18] Tang J-L Liu B-Y Ma K-W. Traditional chinese medicine. Lancet. (2008) 372:1938–40. doi: 10.1016/S0140-6736(08)61354-9 18930523

[19] Borchers AT Hackman RM Keen CL Stern JS Gershwin ME. Complementary medicine: a review of immunomodulatory effects of Chinese herbal medicines. Am J Clin Nutr. (1997) 66:1303–12. doi: 10.1093/ajcn/66.6.1303 9394679

[20] Jiang M-H Zhu L Jiang J-G. Immunoregulatory actions of polysaccharides from Chinese herbal medicine. Expert Opin Ther Targets. (2010) 14:1367–402. doi: 10.1517/14728222.2010.531010 21058924

[21] Huang C-F Lin S-S Liao P-H Young S-C Yang C-C. The immunopharmaceutical effects and mechanisms of herb medicine. Cell Mol Immunol. (2008) 5:23–31. doi: 10.1038/cmi.2008.3 18318991 PMC4652916

[22] Borchers AT Sakai S Henderson GL Harkey MR Keen CL Stern JS. Shosaiko-to and other Kampo (Japanese herbal) medicines: a review of their immunomodulatory activities. J Ethnopharmacol. (2000) 73:1–13. doi: 10.1016/s0378-8741(00)00334-2 11025134

[23] Broadus RN. Toward a definition of “bibliometrics. Scientometrics. (1987) 12:373–9. doi: 10.1007/BF02016680

[24] Wang S Zhou H Zheng L Zhu W Zhu L Feng D. Global trends in research of macrophages associated with acute lung injury over past 10 years: A bibliometric analysis. Front Immunol. (2021) 12:669539. doi: 10.3389/fimmu.2021.669539 34093568 PMC8173163

[25] Zhao J Liu G Tao C. Hotspots and future trends of autophagy in Traditional Chinese Medicine: A Bibliometric analysis. Heliyon. (2023) 9:e20142. doi: 10.1016/j.heliyon.2023.e20142 37780780 PMC10539644

[26] Ma L Ma J Teng M Li Y. Visual analysis of colorectal cancer immunotherapy: A bibliometric analysis from 2012 to 2021. Front Immunol. (2022) 13:843106. doi: 10.3389/fimmu.2022.843106 35432385 PMC9009266

[27] Shen J Shen H Ke L Chen J Dang X Liu B. Knowledge mapping of immunotherapy for hepatocellular carcinoma: A bibliometric study. Front Immunol. (2022) 13:815575. doi: 10.3389/fimmu.2022.815575 35173728 PMC8841606

[28] Peng Z Xiao H Tan Y Zhang X. Spotlight on macrophage pyroptosis: A bibliometric and visual analysis from 2001 to 2023. Heliyon. (2024) 10:e31819. doi: 10.1016/j.heliyon.2024.e31819 38845992 PMC11154638

[29] Wang S Zhang L Jin Z Wang Y Zhang B Zhao L. Visualizing temporal dynamics and research trends of macrophage-related diabetes studies between 2000 and 2022: a bibliometric analysis. Front Immunol. (2023) 14:1194738. doi: 10.3389/fimmu.2023.1194738 37564641 PMC10410279

[30] Ye L Fan S Zhao P Wu C Liu M Hu S. Potential herb–drug interactions between anti-COVID-19 drugs and traditional Chinese medicine. Acta Pharm Sin B. (2023) 13:3598–637. doi: 10.1016/j.apsb.2023.06.001 PMC10239737 37360014

[31] Huang K Zhang P Zhang Z Youn JY Wang C Zhang H. Traditional Chinese Medicine (TCM) in the treatment of COVID-19 and other viral infections: Efficacies and mechanisms. Pharmacol Ther. (2021) 225:107843. doi: 10.1016/j.pharmthera.2021.107843 33811957 PMC8011334

[32] Hussain S Xie Y-J Li D Malik SI Hou J Leung EL-H. Current strategies against COVID-19. Chin Med. (2020) 15:70. doi: 10.1186/s13020-020-00353-7 32665783 PMC7344049

[33] Chen Z Nakamura T. Phytotherapy research. Medicinal Chem J. (2004) 18:592–4. doi: 10.1002/ptr.1485 15305324

[34] Lau TF Leung PC Wong ELY Fong C Cheng KF Zhang SC. Using herbal medicine as a means of prevention experience during the SARS crisis. Am J Chin Med. (2005) 33:345–56. doi: 10.1142/S0192415X05002965 16047553

[35] Li M Wu Y Qiu J Lei J Li M Xu N. Huangqin Decoction ameliorates ulcerative colitis by regulating fatty acid metabolism to mediate macrophage polarization via activating FFAR4-AMPK-PPARα pathway. J Ethnopharmacology. (2023) 311:116430. doi: 10.1016/j.jep.2023.116430 36997133

[36] Li J Wang S Wang N Zheng Y Yang B Wang X. Aiduqing formula inhibits breast cancer metastasis by suppressing TAM/CXCL1-induced Treg differentiation and infiltration. Cell Commun Signal. (2021) 19:89. doi: 10.1186/s12964-021-00775-2 34461944 PMC8404313

[37] Yang Y Sun M Yao W Wang F Li X Wang W. Compound kushen injection relieves tumor-associated macrophage-mediated immunosuppression through TNFR1 and sensitizes hepatocellular carcinoma to sorafenib. J Immunother Cancer. (2020) 8:e000317. doi: 10.1136/jitc-2019-000317 32179631 PMC7073790

[38] Long J Liu X-K Kang Z-P Wang M-X Zhao H-M Huang J-Q. Ginsenoside Rg1 ameliorated experimental colitis by regulating the balance of M1/M2 macrophage polarization and the homeostasis of intestinal flora. Eur J Pharmacol. (2022) 917:174742. doi: 10.1016/j.ejphar.2022.174742 34999087

[39] Li Y-Y He Y-X Wu Y-Q Liu C Ren L-Z Lu X-Y. Compatibility between cold-natured medicine CP and hot-natured medicine AZ synergistically mitigates colitis mice through attenuating inflammation and restoring gut barrier. J Ethnopharmacology. (2023) 303:115902. doi: 10.1016/j.jep.2022.115902 36395977

[40] Zhang Y Zeng M Zhang X Yu Q Wang L Zeng W. Tiaogan daozhuo formula attenuates atherosclerosis via activating AMPK -PPARγ-LXRα pathway. J Ethnopharmacology. (2024) 324:117814. doi: 10.1016/j.jep.2024.117814 38286155

[41] Jin Z Luo Y Zhao H Cui J He W Li J. Qingre Huoxue Decoction regulates macrophage polarisation to attenuate atherosclerosis through the inhibition of NF-κB signalling-mediated inflammation. J Ethnopharmacology. (2023) 301:115787. doi: 10.1016/j.jep.2022.115787 36206868

[42] Chen Q Qi X Zhang W Zhang Y Bi Y Meng Q. Catalpol inhibits macrophage polarization and prevents postmenopausal atherosclerosis through regulating estrogen receptor alpha. Front Pharmacol. (2021) 12:655081. doi: 10.3389/fphar.2021.655081 33995075 PMC8120111

[43] Luo Y Sun G Dong X Wang M Qin M Yu Y. Isorhamnetin attenuates atherosclerosis by inhibiting macrophage apoptosis via PI3K/AKT activation and HO-1 induction. PloS One. (2015) 10:e0120259. doi: 10.1371/journal.pone.0120259 25799286 PMC4370599

[44] Gao S Liu Z Li H Little PJ Liu P Xu S. Cardiovascular actions and therapeutic potential of tanshinone IIA. Atherosclerosis. (2012) 220:3–10. doi: 10.1016/j.atherosclerosis.2011.06.041 21774934

[45] Tian L Chen J Yang M Chen L Qiu J Jiang Y. Xiezhuo Tiaozhi formula inhibits macrophage pyroptosis in the non-alcoholic fatty liver disease by targeting the SIRT1 pathway. Phytomedicine. (2024) 131:155776. doi: 10.1016/j.phymed.2024.155776 38851104

[46] Zheng Y Ji S Li X Wen L. Qijia rougan formula ameliorates ECM deposition in hepatic fibrosis by regulating the JAK1/STAT6-*microRNA-23a* feedback loop in macrophage M2 polarization. Biomedicine Pharmacotherapy. (2023) 168:115794. doi: 10.1016/j.biopha.2023.115794 37922651

[47] Fan X Mai C Zuo L Huang J Xie C Jiang Z. Herbal formula BaWeiBaiDuSan alleviates polymicrobial sepsis-induced liver injury via increasing the gut microbiota Lactobacillus johnsonii and regulating macrophage anti-inflammatory activity in mice. Acta Pharm Sin B. (2023) 13:1164–79. doi: 10.1016/j.apsb.2022.10.016 PMC10031256 36970196

[48] Yunna C Mengru H Lei W Weidong C. Macrophage M1/M2 polarization. Eur J Pharmacol. (2020) 877:173090. doi: 10.1016/j.ejphar.2020.173090 32234529

[49] Mantovani A Sica A Sozzani S Allavena P Vecchi A Locati M. The chemokine system in diverse forms of macrophage activation and polarization. Trends Immunol. (2004) 25:677–86. doi: 10.1016/j.it.2004.09.015 15530839

[50] Luo M Zhao F Cheng H Su M Wang Y. Macrophage polarization: an important role in inflammatory diseases. Front Immunol. (2024) 15:1352946. doi: 10.3389/fimmu.2024.1352946 38660308 PMC11039887

[51] Li W You L Lin J Zhang J Zhou Z Wang T. An herbal formula Shenlian decoction upregulates M1/M2 macrophage proportion in hepatocellular carcinoma by suppressing complement cascade. Biomedicine Pharmacotherapy. (2024) 177:116943. doi: 10.1016/j.biopha.2024.116943 38878636

[52] An L Lu M Xu W Chen H Feng L Xie T. Qingfei oral liquid alleviates RSV-induced lung inflammation by promoting fatty-acid-dependent M1/M2 macrophage polarization via the Akt signaling pathway. J Ethnopharmacol. (2022) 298:115637. doi: 10.1016/j.jep.2022.115637 35970312

[53] Li S Zhang B. Traditional Chinese medicine network pharmacology: theory, methodology and application. Chin J Natural Medicines. (2013) 11:110–20. doi: 10.1016/S1875-5364(13)60037-0 23787177

[54] Wei Y Fan Y Ga Y Zhang Y Han J Hao Z. Shaoyao decoction attenuates DSS-induced ulcerative colitis, macrophage and NLRP3 inflammasome activation through the MKP1/NF-κB pathway. Phytomedicine. (2021) 92:153743. doi: 10.1016/j.phymed.2021.153743 34583225

[55] Wang Y Wang X Li Y Xue Z Shao R Li L. Xuanfei Baidu Decoction reduces acute lung injury by regulating infiltration of neutrophils and macrophages via PD-1/IL17A pathway. Pharmacol Res. (2022) 176:106083. doi: 10.1016/j.phrs.2022.106083 35033647 PMC8757644

[56] Cheng P Tang H Dong Y Liu K Jiang P Liu Y. Knowledge mapping of research on land use change and food security: A visual analysis using citespace and VOSviewer. Int J Environ Res Public Health. (2021) 18:13065. doi: 10.3390/ijerph182413065 34948674 PMC8701921

[57] Merigó JM Yang J-B. A bibliometric analysis of operations research and management science. Omega. (2017) 73:37–48. doi: 10.1016/j.omega.2016.12.004

[58] van Eck NJ Waltman L. Software survey: VOSviewer, a computer program for bibliometric mapping. Scientometrics. (2010) 84:523–38. doi: 10.1007/s11192-009-0146-3 PMC2883932 20585380

